# Psychometric evaluation of the Clinical Interview for Externalizing Disorders (ILF-EXTERNAL) in an online setting – Results from the consortium project INTEGRATE-ADHD

**DOI:** 10.25646/12536

**Published:** 2024-09-18

**Authors:** Sophia Weyrich, Vanessa Scholz, Leila Hetzke, Sanna Ulsamer, Chantal Wallau, Diana Mager, Julia Geißler, Marcel Romanos, Ann-Kristin Beyer, Robert Schlack, Anne Kaman, Ulrike Ravens-Sieberer, Julian Witte, Anna Grau, Anna Horn, Peter Heuschmann, Cordula Riederer, the INTEGRATE-ADHD Study Group, Thomas Jans

**Affiliations:** 1 University Hospital Würzburg, Centre of Mental Health, Department of Child and Adolescent Psychiatry, Psychosomatics and Psychotherapy, Würzburg, Germany; 2 Robert Koch Institute, Department of Epidemiology and Health Monitoring, Berlin, Germany; 3 University Medical Centre Hamburg-Eppendorf, Department of Child and Adolescent Psychiatry, Psychotherapy and Psychosomatics, Research Section ‘Child Public Health’, Hamburg, Germany; 4 Vandage GmbH, Bielefeld, Germany; 5 University of Würzburg, Institute of Clinical Epidemiology and Biometry, Würzburg, Germany; 6 University Hospital Würzburg, Clinical Trial Centre, Würzburg, Germany; 7 University Hospital Würzburg, Institute for Medical Data Sciences, Würzburg, Germany; 8 DAK-Gesundheit, Hamburg, Germany

**Keywords:** Children, adolescents, ADHD, Attention-deficit/hyperactivity disorder, clinical interview, ILF-EXTERNAL, reliability, validity, online diagnostics, psychometrics, psychometric properties

## Abstract

**Background:**

The study examines the psychometric properties of the ADHD section of the semi-structured diagnostic interview ILF-EXTERNAL, which was conducted online via video chat.

**Methods:**

As part of the INTEGRATE-ADHD research project, 202 children and adolescents (age *M* = 12.87 years, *SD* = 3.04, 28.2 % female) with an administrative diagnosis of ADHD registered with their health insurance company were clinically assessed for the presence of ADHD according to the German ADHD S3 guideline. Using the ILF-EXTERNAL, one parent and, from the age of eight, also the children themselves were interviewed. A proxy rating by a parent was made using the German FBB-ADHS rating scale. In a subsample (*n* = 65), an independent blind interviewer rated the videorecordings of the ILF-EXTERNAL parent interview to determine the interrater reliability of the ILF-EXTERNAL.

**Results:**

All ADHD symptom scales of the ILF-EXTERNAL showed good to excellent internal consistency (α = 0.89 to 0.93). Interrater reliability was high for both categorical and dimensional analyses (κ = 0.78 and κ = 0.81; ICC(1,1) = 0.97 and 0.98). High correlations of the ILF-EXTERNAL parent interview with the FBB-ADHS (*r* = 0.79 to *r* = 0.85) and with the ILF-EXTERNAL child interview (*r* = 0.60 to *r* = 0.71) demonstrated convergent validity.

**Conclusions:**

Sound psychometric properties of the ILF-EXTERNAL were also confirmed for its use in an online setting. High interrater reliabilities demonstrate the quality of the ADHD diagnostics carried out in the consortium project INTEGRATE-ADHD.

## 1. Introduction

Attention-deficit/hyperactivity disorder (ADHD) is characterised by the core symptoms of inattention, hyperactivity and impulsivity [1]. Diagnosis requires the onset of symptoms in childhood, the presence of symptoms in two or more settings for at least six months, and a level of severity that causes functional impairment or psychological strain in the individual [2]. It must be excluded that the symptoms are better explained by another mental disorder [2]. ADHD is diagnosed following a detailed multimodal clinical assessment that takes into account current diagnostic criteria (DSM-5 [2], ICD-10 [3]) and assessment standards according to established guidelines [4–7]. The ICD-11 diagnostic criteria for ADHD [8], which will be used after a transition period, are largely congruent with those of the DSM-5.

A comprehensive clinical ADHD assessment is a central component of the INTEGRATE-ADHD research project. In this project, which is funded by the Innovation Fund of the German Federal Joint Committee (Gemeinsamer Bundesausschuss, G-BA), parents of children and adolescents for whom an ADHD diagnosis is administratively documented by their health insurance company are asked about their child’s ADHD diagnosis using questionnaires from the German Health Interview and Examination Survey for Children and Adolescents (KiGGS) [9]. A subsample will be examined using a clinical ADHD assessment according to the ADHD S3 guideline of the Association of the Scientific Medical Societies in Germany (Arbeitsgemeinschaft der Wissenschaftlichen Medizinischen Fachgesellschaften e.V., AWMF) [4], with the aim of clinically validating and thus better interpreting prevalence estimates from administrative and epidemiological data sources (for background information, see the editorial by Schlack and Romanos in issue 3/2024 of the Journal of Health Monitoring [10]). A special feature of the clinical ADHD assessment in this project is that it was conducted entirely in an online setting. The online setting was used due to the contact restrictions in the context of the COVID-19 pandemic. Moreover, online diagnostics is a modern concept with many advantages. For example, the flexibility of online diagnostics in terms of time and place saves time for both the patients and their clinicians. Especially in rural areas with difficult access to healthcare and public transport, online diagnostics can be a good option. A detailed description of the ADHD assessment carried out in an online setting as part of INTEGRATE-ADHD as well as a discussion of the advantages and disadvantages of online diagnostics can be found in the article by Hetzke et al. [11].

To obtain information in the diagnostic process, established ADHD guidelines recommend the use of multiple diagnostic methods and multiple informants (e.g. interviews with the individual and their caregivers, self- and proxy-rating scales, behavioural observation, performance tests) [4–7]. A clinical diagnosis is then made on the basis of all the information gathered [4–7]. The clinical interview is the gold standard of the clinical examination for questioning the person and their caregivers [1, 12]. In addition to the disorder-specific medical history and psychosocial situation, the diagnostic criteria defined in DSM-5 [2] and ICD-10 [3] should be recorded [13]. Ideally, (semi-)structured diagnostic interviews are used [1, 13] to ensure that ADHD symptoms are asked in a targeted and structured manner according to the diagnostic criteria. Although clinical interviews are considered essential in the diagnostic process, ADHD guidelines suggest that they should always be supplemented by other diagnostic methods [4–7].

An important diagnostic tool of the clinical assessment in the INTEGRATE-ADHD research project was the Clinical Interview for Externalizing Disorders (Interview-Leitfaden für Externale Störungen, ILF-EXTERNAL) from the Diagnostic System for Mental Disorders based on DSM-5 for Children and Adolescents (Interview-Leitfäden zum Diagnostik-System für psychische Störungen nach DSM-5 für Kinder und Jugendliche, DISYPS-ILF) [14]. To date, only data from the multicentre research project Evidence-based, Stepped Care of ADHD school (ESCAschool) [15] are available on the psychometric properties of the ILF-EXTERNAL. Under the leadership of the DISYPS-ILF group of authors, the ILF-EXTERNAL was evaluated in a clinical sample of 474 children with ADHD symptoms aged 6 to 12 years. Overall, the ILF-EXTERNAL proved to be a reliable and valid measure [14, 16]. To date, there is a complete lack of data on the psychometric properties for the implementation of the ILF-EXTERNAL in an online setting. The present study aims to fill this gap.

The ILF-EXTERNAL is a semi-structured diagnostic interview for the assessment of externalizing disorders (ADHD and conduct disorder) in children and adolescents that can be conducted with the parents and, from the age of eight, with the child or adolescent [14]. Unlike fully structured interviews, the ILF-EXTERNAL allows for flexible questioning by the interviewer. In addition to information from the interview, the evaluation of the patient’s behaviour during the interview can be included in the rating [14]. The pre-written questions do not have to be asked literally, so it is possible to adapt the questions to the developmental level of the child or to include individual expressions used by the child or parents to increase comprehensibility [14]. As part of a multimodal diagnostic approach, the ILF-EXTERNAL can be combined with diagnostic instruments from the German Diagnostic System of Mental Disorders in Children and Adolescents based on the ICD-10 and DSM-5 – III (Diagnostik-System für Psychische Störungen nach ICD-10 und DSM-5 für Kinder und Jugendliche – III, DISYPS-III) [17] (e.g. rating scales for proxy- and self-report) [14]. The interview corresponds in terminology and dimensions to the corresponding DISYPS-III rating scales and are therefore particularly suitable for cross-method validity studies.

As part of the INTEGRATE-ADHD project, 202 children and adolescents with an administrative diagnosis of ADHD were clinically assessed for the presence of ADHD according to the AWMF S3 guideline [4]. One parent and the children and adolescents were interviewed using the ILF-EXTERNAL via video chat. In addition, ADHD rating scales from the DISYPS-III were completed online.

The aim of this study was to analyse the psychometric properties of the ADHD section of the ILF-EXTERNAL conducted with a parent. This will contribute to the quality assurance of ADHD diagnostics within the INTEGRATE-ADHD project. In addition, the empirical basis for the psychometric properties of the ILF-EXTERNAL was to be extended by analysing a sample with a wider age range and greater variability of ADHD symptoms than previously available [14, 16]. A special feature of the INTEGRATE-ADHD project was the implementation of the ILF-EXTERNAL in an online setting. An investigation of the psychometric properties found in this setting has not yet been conducted. The psychometric properties determined in this study were to be compared with those determined in a face-to-face implementation [14, 16]. We analysed descriptive statistics and reliability measures for internal consistency and interrater reliability as well as convergent validity of the ADHD section of the ILF-EXTERNAL parent interview.

## 2. Methods

### 2.1 Procedure and sample

All parents of the children and adolescents examined took part in the online survey of the consortium project INTEGRATE-ADHD. The target group consisted of children and adolescents who were insured with the German statutory health insurance company DAK-Gesundheit in 2020 and who had a confirmed administrative diagnosis of ADHD (ICD-10: F90.0-9) in at least one quarter of 2020 (M1Q criterion). The clinical ADHD assessment was carried out in 202 children and adolescents together with at least one parent. Inclusion criteria for the clinical assessment were age between 3 and 17 years (in 2020) and informed consent to participate in the assessment (signed by the parent or legal guardian and, from the age of eight, also by the child). As the analyses planned in the INTEGRATE-ADHD project required the comparison of two equally large groups of children and adolescents with and without a parent-reported diagnosis of ADHD in the online survey, the sample for the clinical assessment was drawn from the online sample using a stratified random procedure. The clinical assessments were carried out between January 2022 and January 2023. A detailed description of the INTEGRATE-ADHD research project can be found in Schlack et al. [18] and Beyer et al. [19].

The clinical assessments were conducted by one of seven specially trained psychologists or psychotherapists in education at the Department of Child and Adolescent Psychiatry, Psychosomatics and Psychotherapy at the University Hospital of Würzburg. The diagnosticians were part of the INTEGRATE-ADHD research project and were informed about the composition of the study sample. In addition to the ADHD section of the ILF-EXTERNAL, which was administered to one or both parents and, from the age of eight, also to the child, other diagnostic methods were used in accordance with the German AWMF S3 guideline [4] (medical history interview, proxy- and self-rating scales, behavioural observation, performance tests (attention test, intelligence test) and review of previous medical reports and school reports). The interviews, behavioural observation and performance tests were conducted via video chat (Skype for Business). This took three to four hours per appointment with one parent and the child, and was recorded on video and audio. In addition, participants completed questionnaires as part of an online survey. A detailed description of the clinical ADHD assessment and its implementation in an online setting can be found in the article by Hetzke et al. [11].

**ADHD in Germany – Comparison and integration of administrative and epidemiological ADHD diagnostic data through clinical assessment (INTEGRATE-ADHD)**

**Consortium partners:** Robert Koch Institute Berlin, Department of Epidemiology and Health Monitoring, Germany; University Hospital Würzburg, Department of Child and Adolescent Psychiatry, Psychosomatics and Psychotherapy, Germany; University Medical Centre Hamburg-Eppendorf, Department of Child and Adolescent Psychiatry, Psychotherapy and Psychosomatics, Research Section ‘Child Public Health’, Germany; Vandage GmbH, Germany; University of Würzburg, Germany, Institute for Clinical Epidemiology and Biometry, Germany; DAK-Gesundheit, Germany

**Data holder:** Robert Koch Institute

**Objectives:** Identification of potential causes for the discrepancies between administrative ADHD diagnostic data (based on health insurance claims data) and epidemiological ADHD diagnostic data (based on surveys) for Germany, integration and validation of these data through a guideline-based clinical examination

**Study design:** Cross-sectional online survey, additional clinical examination of a sub-sample, data linkage with administrative health insurance data

**Population:** Children and adolescents who were insured with DAK-Gesundheit in 2020 and who were 0 to 17 years old at that time and for whom an administrative ADHD diagnosis labelled as confirmed was available in at least one quarter

**Gross sample**: 24,880 children and adolescents insured with DAK-Gesundheit with an administrative ADHD diagnosis

**Net sample:** 5,461 surveyed parents, 202 clinically examined children and adolescents

**Data collection period:** October 2021 to August 2022 (online survey), January 2022 to January 2023 (online clinical examination)

More information in German at www.rki.de/integrate-adhd

In order to determine the interrater reliability (the level of agreement between two or more raters) of the ADHD interview (ILF-EXTERNAL), a sub-sample of 65 participants was randomly selected after the completion of the clinical assessments, and the corresponding videotape of the parent interview was randomly assigned to an independent diagnostician who was blinded to the previous interview results. At this point in the project, five of the original seven diagnosticians were still available for assignment. To determine interrater reliability, only participants for whom a video recording of the parent interview was available in sufficient quality were included. The subsample was thus selected from a total of 153 out of 202 clinically examined children and adolescents by project staff from the Institute for Clinical Epidemiology and Biometry at the University of Würzburg, who were not involved in the clinical assessments and were not informed on the individual examination results. This ensured random selection and random assignment to the second diagnostician. The second diagnostician then rated the parents’ reports in the ILF-EXTERNAL on the basis of the video recordings (in the following, she or he will be referred to as the second rater).

Interrater reliability was determined using intraclass correlation (ICC) [20, 21]. Sample size was determined as part of an a-priori-power-analysis based on the recommendations of Zou [22] and the R-package ICC.Sample.Size. According to this, a sample size of at least 61 cases was required so that the lower limit of a one-sided 95 % confidence interval of an ICC of 0.80 with an 80 % confidence interval is not less than 0.65.

### 2.2 Measurement methods

The ILF-EXTERNAL measures both ADHD and conduct disorder [14]. As the consortium project INTEGRATE-ADHD focused on the diagnosis of ADHD, only the ADHD section of the ILF-EXTERNAL was used and examined in the present study. This will be referred to as ILF-EXTERNAL-ADHD.

The ILF-EXTERNAL-ADHD is divided into the symptom scales inattention and hyperactivity/impulsivity with nine items each (according to the nine ADHD symptom criteria of the DSM-5), which can be combined to form a total ADHD symptom scale, and the scale functional impairment with five items. Each item is to be rated by the diagnostician on a four-point Likert scale from 0 (not (noticeable) present or age-typical) to 3 (very pronounced). For the latter rating, an example of a child’s behaviour representing a rating of 3 is provided. The symptom criterion is clearly defined for each item. In addition, there are notes on its differentiation from similar symptoms from the ADHD- and conduct disorder-section of the ILF-EXTERNAL. Several examples of questions are suggested for each symptom criterion. These can be adapted, so that the diagnostician is able to flexibly explore the symptoms. Both a categorical evaluation to establish a clinical diagnosis (meeting the minimum number of symptom criteria required for a diagnosis according to DSM-5 or ICD-10 and meeting the additional criteria) and a dimensional evaluation to assess severity (mean item scores of the scales) can be performed [14]. The ILF-EXTERNAL-ADHD will also be applicable for the diagnosis of ADHD according to ICD-11 [8], as its ADHD diagnostic criteria largely correspond to those of DSM-5.

Semi-structured interviews should be conducted by clinically experienced diagnosticians [23]. The diagnosticians in the project were psychologists or psychotherapists in education. They received extensive training in the use of the ILF-EXTERNAL-ADHD. After training, the diagnosticians’ ratings were not allowed to deviate significantly from an expert consensus standard (see Hetzke et al. [11]). If the participating children and adolescents were taking medication for ADHD, the diagnosticians were asked to assess the current severity of symptoms without the effect of medication.

For a proxy rating of the child’s ADHD symptoms by a parent, the German Symptom Checklist for Attention-deficit/Hyperactivity Disorder (Fremdbeurteilungsbogen für Aufmerksamkeitsdefizit-/Hyperaktivitätsstörungen, FBB-ADHS or FBB-ADHS-V for preschool children) from the DISYPS-III [17] was used, whose scales and items correspond to those of the ILF-EXTERNAL-ADHD.

### 2.3 Statistical analyses

The statistical analyses described below were carried out using the IBM SPSS Statistics program (version 29.0.0.0).

Descriptive statistics were determined for the mean item scores of the ILF-EXTERNAL-ADHD scales and, as part of an item analysis, item discriminative power (corrected item-total correlation) was determined for the individual items.

Cronbach’s alpha [24] was calculated as a reliability measure to determine the internal consistency of the scales.

Cohen’s kappa was calculated to determine the interrater reliability between the diagnostician and the second rater in a categorical evaluation of the ILF-EXTERNAL-ADHD (symptom criteria required for diagnosis according to ILF-EXTERNAL-ADHD fulfilled or not fulfilled). No differentiation was made according to the diagnostic subtypes, i.e. it was only recorded whether the symptom criteria for any diagnosis were fulfilled or not (DSM-5: ADHD with combined, with predominantly inattentive or with predominantly hyperactive-impulsive symptoms; ICD-10: Attention-deficit/hyperactivity disorder or attention deficit disorder without hyperactivity). The intraclass correlation (ICC) was calculated to determine the interrater reliability in the dimensional evaluation of the ILF-EXTERNAL-ADHD scales. The one way random effects absolute agreement model for single rater/measurements (ICC(1,1)) was used, as the original interview and the second rating were always conducted by a different one of a total of seven diagnosticians and the absolute agreement between the ratings and the values of the individual raters were relevant [20, 21, 25, 26].

To determine the convergent validity of the ILF-EXTERNAL-ADHD, product-moment correlations were calculated between the scales of the ILF-EXTERNAL-ADHD conducted with a parent and the corresponding scales of the FBB-ADHS rating scale completed by a parent as well as the scales of the ILF-EXTERNAL-ADHD conducted with the child. In addition, the categorical agreement of the ILF-EXTERNAL-ADHD parent interview with the FBB-ADHS parent rating scale was determined using the Phi coefficient (number of symptom criteria required for diagnosis according to DSM-5 fulfilled or not fulfilled).

## 3. Results

### 3.1 Sample

Age and gender of the children and adolescents are shown in Table 1 for the total sample of all children and adolescents who participated in the clinical examination (*N* = 202), as well as for the 65 randomly selected for interrater reliability and the 137 not selected. There were no meaningful differences between the subgroups.

The ILF-EXTERNAL-ADHD interview was conducted with both father and mother in 16 children and adolescents, with the mother only in 170, and with the father only in 11. In exceptional cases (*n* = 5), the ILF-EXTERNAL-ADHD was conducted with another caregiver with whom the child was living (e.g. grandparents).

In a categorical evaluation of the ILF-EXTERNAL-ADHD conducted with a parent (or another caregiver), 97 of the 202 children and adolescents met the DSM-5 symptom criteria for ADHD (A criterion). Most had ADHD with predominantly inattentive symptoms (Table 1). The ILF-EXTERNAL-ADHD is an important, but not sufficient basis for a clinical diagnosis of ADHD as part of the clinical assessment in the INTEGRATE-ADHD project (see also the article by Hetzke et al. [11]). Extensive analyses of the discrepancy between administratively documented and clinical ADHD diagnoses have been carried out and will be published elsewhere.

### 3.2 Mean item scores and item analysis

Descriptive statistics for the mean item scores of the ILF-EXTERNAL-ADHD scales conducted with a parent are presented in Table 2. The mean scores ranged from *M* = 0.88 (*SD* = 0.68) for the functional impairment scale to *M* = 1.38 (*SD* = 0.71) for the inattention scale. With a range of *r* = 0.46 to 0.69, the discriminative power of all items was in a good range for all items.

### 3.3 Reliability – internal consistency

The results of the reliability analysis of the individual scales of the ILF-EXTERNAL-ADHD are presented in Table 2. All symptom scales of the ILF-EXTERNAL-ADHD showed good to excellent internal consistency with a Cronbach’s alpha of α = 0.89 to 0.93, both when conducted with a parent and when conducted with the child. With a Cronbach’s alpha of α = 0.70 (child) and 0.79 (parent), the functional impairment scale was within an acceptable range. As the interview was only conducted with the child from the age of eight (*n* = 188), and with sufficient cooperation from the child, the sample in these analyses was reduced from 178 to 182 children and adolescents, depending on the scale, due to missing data.

### 3.4 Interrater reliability

The interrater reliability for categorical evaluation of the ILF-EXTERNAL-ADHD (symptom criteria required for diagnosis according to ILF-EXTERNAL-ADHD fulfilled or not fulfilled) between the diagnostician and the second rater was in the range of substantial agreement with a Cohen’s kappa of κ = 0.78 (*p* < 0.001) for diagnosis according to DSM-5 and in the range of almost perfect agreement according to ICD-10 with a Cohen’s kappa of κ = 0.81 (*p* < 0.001) [27]. A cross-tabulation of agreement between the diagnostician and the second rater is shown in Table 3. For 58 of the 65 children and adolescents, the categorical evaluation of the original diagnostician and the second rater were in agreement, giving a rate of agreement of 89.2 %.

The intraclass correlations for dimensional interrater reliability are shown in Table 2. With ICC coefficients of ICC(1,1) = 0.97 and 0.98, all ICCs of the ILF-EXTERNAL-ADHD scales showed excellent interrater reliability [28, 29].

**Figure 1:**
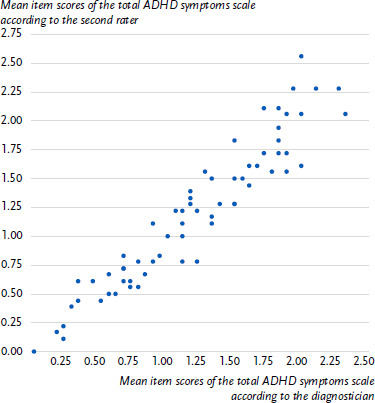
Mean item scores of the total ADHD symptoms scale (ILF-EXTERNAL): Rating by the original diagnostician and the second rater (*n* = 65). Source: INTEGRATE-ADHD, clinical dataset ADHD = attention-deficit/hyperactivity disorder

A scatterplot showing the mean item scores of the original diagnostician and the second rater on the ILF-EXTERNAL total ADHD symptoms scale is shown in Figure 1.

### 3.5 Convergent validity

Table 4 shows the convergent validity results. The correlations of the scales of the ILF-EXTERNAL-ADHD conducted with a parent with the corresponding scales of the FBB-ADHS and FBB-ADHS-V rating scales ranged from *r* = 0.79 to *r* = 0.85 and thus showed a high correlation. In 199 children and adolescents, the same parent (or other caregiver) was always involved in both ILF-EXTERNAL-ADHD and FBB-ADHS. In three children and adolescents the parents were different. The means of the total ADHD symptoms scale for ILF-EXTERNAL-ADHD (*M* = 1.16, *SD* = 0.63) and FBB-ADHD (*M* = 1.24, *SD* = 0.63) were not meaningfully different. Looking at categorical agreement (number of DSM-5 symptom criteria fulfilled or not fulfilled), the ILF-EXTERNAL and the FBB-ADHS yielded the same result in 173 out of 202 cases, that is a rate of agreement of 85.6 %. A phi coefficient of φ = 0.71 (*p* < 0.001) also indicates a high correlation.

The correlations of the scales of the ILF-EXTERNAL-ADHD conducted with a parent with the corresponding scales of the ILF-EXTERNAL-ADHD conducted with the child were between *r* = 0.60 and *r* = 0.71 and thus also showed a high correlation. As the interview was only conducted with the child from the age of eight (*n* = 188) and with sufficient cooperation from the child, the sample in these analyses was reduced to between 178 and 182 participants (number depending on the scale) due to missing data.

## 4. Discussion

The aim of this study was to analyse the psychometric properties of the ADHD section of the ILF-EXTERNAL [14], which was conducted with a parent in an online setting. This was for the quality assurance of diagnostics within the framework of the INTEGRATE-ADHD research project. In addition, the present study contributes to expanding the empirical basis of the psychometric properties of the ILF-EXTERNAL, as only a few studies have been conducted on this diagnostic tool. In particular, there are no studies on the implementation of the ILF-EXTERNAL in an online setting.

The results of this study confirm the good psychometric properties of the ADHD section of the ILF-EXTERNAL, even when conducted online via video chat. The internal consistencies of all symptom scales were good to excellent. Convergent validity was demonstrated by high correlations with the ILF-EXTERNAL child interview and the FBB-ADHS rating scale [17]. High interrater reliability were found for the ILF-EXTERNAL-ADHD, which underlines the objectivity of the ILF-EXTERNAL-ADHD and contributes to the quality assurance of the ADHD diagnostics in the INTEGRATE-ADHD project.

To date, the psychometric properties of the ILF-EXTERNAL have only been analysed by ILF-EXTERNAL authors and co-workers in the sample of the ESCAschool research project [15]. This clinical sample consisted of 474 children aged 6 to 12 years with ADHD symptoms [14, 16] who wished to participate in a treatment trial. In contrast, the sample in the present study (*N* = 202) represented a broader age range, from 5 to 19 years (*M* = 12.87, *SD* = 3.04). Although each of our participating children and adolescents had an administrative diagnosis of ADHD, only about half of the participating parents had reported the ADHD diagnosis in the online survey. Furthermore, because our sample was not pre-selected on the basis of a request for treatment, we expected a lower severity and greater variability of ADHD symptoms compared to the ESCAschool sample, which was confirmed: The mean scores of the ILF-EXTERNAL-ADHD scales in our sample ranged from *M* = 0.88 (*SD* = 0.68) to *M* = 1.38 (*SD* = 0.71) and were thus lower than in the sample of the ILF-EXTERNAL authors’ group, in which the mean scores for the ADHD section scales ranged from *M* = 1.61 (*SD* = 0.59) to *M* = 1.95 (*SD* = 0.48) [14, 16]. As expected, the standard deviations of the ILF-EXTERNAL-ADHD scales were slightly higher in our sample (*SD* = 0.63 to *SD* = 0.72) than in the ESCAschool sample (*SD* = 0.48 to *SD* = 0.73) [14, 16]. Thus, the analysis of the ILF-EXTERNAL-ADHD in our sample, with its different distribution of age and ADHD severity, is a useful expansion of the data from the ESCAschool sample.

In our study, the items of the ADHD section of the ILF-EXTERNAL showed good discriminative power ranging between *r* = 0.46 and 0.69. The discriminative power of most items was thus higher than measured in the sample of the ILF-EXTERNAL authors’ group (*r* = 0.21 to *r* = 0.68) [14, 16].

The internal consistencies of the symptom scales of the ILF-EXTERNAL-ADHD conducted with a parent (α = 0.89 to 0.93) and the functional impairment scale (α = 0.79) were also slightly higher than in the studies by Görtz-Dorten et al. and Thöne et al. (α = 0.71 to 0.87 for the symptom scales, α = 0.62 for the functional impairment scale) [14, 16]. For methodological reasons, the scale with fewer items (functional impairment) had a lower internal consistency than the symptom scales with more items. When the ILF-EXTERNAL-ADHD was carried out with the child itself, similar internal consistencies were found as when it was conducted with a parent. No data are yet available on the psychometric properties of the ILF-EXTERNAL child interview.

We analysed the interrater reliability of the ILF-EXTERNAL-ADHD using a subsample of 65 children and adolescents. Categorical scoring (ADHD symptom criteria met or not met) resulted in substantial to near perfect interrater reliability coefficients [27]. On a dimensional level, the high level of agreement between the diagnostician and the second rater is impressively shown in the scatterplot (Figure 1). The intraclass correlations showed excellent interrater reliabilities for all scales of the ILF-EXTERNAL-ADHD [28, 29]. These were even slightly higher than the already very high ICCs reported by the ILF-EXTERNAL authors’ group. Görtz-Dorten et al. and Thöne et al. [14, 16] had the ILF-EXTERNAL recordings of 45 participants randomly selected from the ESCAschool sample assessed by two independent raters. They found intraclass correlations from ICC(1,1) = 0.83 to 0.95.

As with Görtz-Dorten et al. und Thöne et al. [14, 16], the second rating to determine interrater reliability was based on video recordings of a previously conducted interview. This is an established procedure for reasons of economy of time. However, a second, completely independent realisation of the interview by the second rater would have been more conservative. It is to be expected, that a second rater would interview the subjects in a different way, and also focus the questions somewhat differently, so that the consistency of the results of the first and second exploration would be lower than with a second rating based on a video recording. Although it was ensured that the second rater did not have access to the results of the first exploration, it could not be guaranteed that the second rater was truly blind to the original diagnostician’s rating. For example, it would have been possible to infer the rating of the diagnostician from the questions he or she asked the parents (e.g. ‘So the forgetfulness is very pronounced?’). In further studies, it would be desirable to analyse interrater reliability on the basis of independent interviews. It should also be noted that the study design was not blinded, i.e. the diagnosticians were aware that the participating children and adolescents had an administrative diagnosis of ADHD. The diagnosticians therefore had prior information about the possible presence of ADHD in the participants, so that a bias in judgement in this respect cannot be ruled out.

To determine convergent validity, in addition to measures of categorical agreement, dimensional correlations of the scales of the ILF-EXTERNAL-ADHD carried out with a parent with the corresponding scales of the FBB-ADHS rating scale were calculated. These showed high correlations, indicating convergent validity. In our sample, these correlations are even higher than in the studies of the ILF-EXTERNAL author’s group (*r* = 0.58 to *r* = 0.78) [14, 16]. We also analysed the correlation between the results of the parent and child interview. Again, our analyses showed high correlations, which can be interpreted as evidence of convergent validity. In evaluating the high correlations between the parent and child interview, it should be noted that the interviews were not conducted independently, but by the same diagnostician. In most cases, the ILF-EXTERNAL was conducted first with the parent and then with the child at a later appointment (sometimes in reverse order, if the family’s schedule did not allow otherwise). This meant that the ILF-EXTERNAL was always carried out in the knowledge of what the parent (or child) had already reported about the ADHD symptoms. In addition, in the case of younger children, a parent was also present during the examination of the child. These circumstances may have contributed to higher agreement between parent and child interviews than it would have been the case with completely independent investigations by different interviewers.

The recently published ILF-EXTERNAL-ADHD, which is based on the current diagnostic criteria for ADHD (DSM-5 [2]), fulfils the quality criteria as well as older semi-structured diagnostic interviews for the assessment of ADHD symptoms based on the diagnostic criteria of the DSM-IV [30] (cf. e.g. the psychometric properties determined by Jans and colleagues [32] for the ADHD scales derived from the Kiddie-SADS [31] from the DSM-IV [30]). The ILF-EXTERNAL can therefore be regarded as a current German-language standard instrument, which is a good replacement for older instruments based on the ADHD diagnostic criteria of the DSM-IV [30] and which can also be used in clinical practice in the future due to the fact that the new ICD-11 [8] ADHD criteria are largely identical to those of the DSM-5.

In conclusion, we were able to demonstrate good psychometric properties of the ILF-EXTERNAL-ADHD. It should be emphasised that these apply to an investigation in an online setting. In our research project INTEGRATE-ADHD, the clinical diagnosis of ADHD is a central endpoint of the investigations. The results of the ADHD diagnostic interview are an important basis for this. The good interrater reliabilities found in our study demonstrate the high quality of data collection in INTEGRATE-ADHD.

## Key statements

- The ADHD section of the Clinical Interview for Externalizing Disorders (ILF-EXTERNAL) also shows sound psychometric properties when conducted in an online setting via video chat.
- The interview can be used not only to make a diagnosis, but also to reliably measure the severity of ADHD symptoms.
- The high interrater reliability demonstrates the high quality of clinical ADHD diagnostics in the INTEGRATE-ADHD research project.

## Figures and Tables

**Table 1:** Description of the sample (*N* = 202, *n* = 57 girls, *n* = 145 boys) of the clinical ADHD assessment as part of the INTEGRATE-ADHD research project. Source: INTEGRATE-ADHD, clinical dataset

	Total sample (*N* = 202)	Drawn for interrater reliability analysis (*n* = 65)	Not drawn for interrater reliability analysis (*n* =137)
**Age at the time of examination**			
*M* (*SD*)	12.87 (3.04)	12.38 (3.26)	13.11 (2.91)
Min/Max	5.58/19.50	5.58/19.17	6.00/19.50
**Gender**			
Female	57 (28.2 %)	18 (27.7 %)	39 (28.5 %)
Male	145 (71.8 %)	47 (72.3 %)	98 (71.5 %)
**ADHD subtype according to ILF-EXTERNAL-ADHD^a^**			
Combined	33 (16.3 %)	13 (20.0 %)	20 (14.6 %)
Predominantly inattentive	54 (26.7 %)	17 (26.1 %)	37 (27.0 %)
Predominantly hyperactive-impulsive	10 (5.0 %)	2 (3.1 %)	8 (5.8 %)

*M* = mean, *SD* = standard deviation, Min = minimum, Max = maximum, ADHD = attention-deficit/hyperactivity disorder, ILF-EXTER NAL = Clinical Interview for Externalizing Disorders (Interview-Leitfaden für Externale Störungen)

^a^Meeting of DSM-5 symptom criteria (A criterion) according to the ILF-EXTERNAL-ADHD conducted with a parent

**Table 2:** Mean item scores, reliability (Cronbach’s alpha) and interrater reliability (intraclass correlation, ICC) of the ILF-EXTERNAL-ADHD scales. Source: INTEGRATE-ADHD, clinical dataset

Scale	Min	Max	*M*	*SD*	Cronbach’s alpha		ICC(1,1) (95 % CI)
	Parent-interview				Parent-interview	Child-interview	Parent-interview
Inattention	0.00^a^	2.89^a^	1.38^a^	0.71^a^	0.89^a^	0.89^b^	0.97^e^ (0.94 – 0.98)
Hyperactivity/impulsivity	0.00^a^	2.89^a^	0.94^a^	0.72^a^	0.89^a^	0.90^b^	0.98^e^ (0.96 – 0.99)
Total ADHD symptoms	0.00^a^	2.56^a^	1.16^a^	0.63^a^	0.91^a^	0.93^c^	0.97^e^ (0.96 – 0.98)
Functional impairment	0.00^a^	2.40^a^	0.88^a^	0.68^a^	0.79^a^	0.70^d^	0.97^e^ (0.95 – 0.98)

^a^*N* = 202,

^b^*n* = 182,

^c^*n* = 181,

^d^*n* = 178,

^e^*n* = 65

Min = minimum, Max = maximum, *M* = mean, *SD* = standard deviation, ICC(1,1) = One way random effects absolute agreement model for single rater/measurements, CI = confidence interval, ADHD = attention-deficit/hyperactivity disorder

**Table 3:** Agreement between diagnostician and second rater on the presence of ADHD according to DSM-5 symptom criteria based on the ILF-EXTERNAL-ADHD administered to a parent (*n* = 65). Source: INTEGRATE-ADHD, clinical dataset

		Second rater		Total
		ADHD	No ADHD	
**Diagnostician**	ADHD	28	4	32
	No ADHD	3	30	33
**Total**		31	34	65

ADHD = attention-deficit/hyperactivity disorder

**Table 4:** Convergent validity: Correlations of the scales of the ILF-EXTERNAL-ADHD conducted with the parent with the scales of the FBB-ADHS ratings by the parent and with the scales of the ILF-EXTERNAL-ADHD conducted with the child. Source: INTEGRATE-ADHD, clinical dataset

Scale	FBB-ADHS parent			ILF-EXTERNAL-ADHD child		
	*r*	95 % CI	*n*	*r*	95 % CI	*n*
Inattention	0.83	0.79 – 0.87	202	0.67	0.58 – 0.74	182
Hyperactivity/impulsivity	0.81	0.76 – 0.85	202	0.69	0.61 – 0.76	182
Total ADHD symptoms	0.85	0.80 – 0.88	202	0.71	0.63 – 0.78	181
Functional impairment	0.79	0.74 – 0.84	202	0.60	0.49 – 0.68	178

*r* = product-moment correlations, CI = confidence interval, ADHD = attention-deficit/hyperactivity disorder, FBB-ADHS = Symptom Checklist for Attentiondeficit/Hyperactivity Disorder (Fremdbeurteilungsbogen für Aufmerksamkeitsdefizit-/Hyperaktivitätsstörungen), ILF-EXTERNAL-ADHD = ADHD section of the Clinical Interview for Externalizing Disorders (Interview-Leitfaden für Externale Störungen)
